# Improved isolation and PCR detection of *Phytophthora agathidicida* oospores from soils

**DOI:** 10.1128/spectrum.00135-25

**Published:** 2025-04-08

**Authors:** Jade T.T. Palmer, Jochem N.A. Vink, Leticia M. Castro, Oliver J.S. Craig, Emily E. Davison, Monica L. Gerth

**Affiliations:** 1 School of Biological Sciences, Victoria University of Wellington 8491, Wellington, New Zealand; USDA-ARS San Joaquin Valley Agricultural Sciences Center, Parlier, California, USA

**Keywords:** *Phytophthora*, oospores, eDNA, PCR, detection, oomycetes, dieback, soil

## Abstract

*Phytophthora* species are eukaryotic microorganisms responsible for severe dieback and root rot in plants worldwide, impacting crops, forests, and other important ecosystems. In New Zealand, *P. agathidicida* leads to fatal dieback in kauri (*Agathis australis*), long-lived endemic trees of significant cultural and ecological importance. A critical aspect of the *P. agathidicida* lifecycle is the production of oospores—thick-walled spores essential for long-term survival in soil, dispersal, and disease inoculation. However, their heterogeneous distribution in soils, robust structure, and dormant state make them challenging to detect using soil baiting or DNA-based methods. Soil baiting is the basis of most current testing for *P. agathidicida*, but baiting-based methods have low sensitivity, are slow, and require specialised facilities. To address these challenges, we developed and validated a PCR-based method for detecting *P. agathidicida* oospores directly from soil. Our approach includes a technique for separating oospores from soil, improved oospore lysis and DNA extraction, and a primer pair that targets a repeat region of the *P. agathidicida* genome with high sensitivity and specificity. The primers amplified the target product in all tested *P. agathidicida* isolates without cross-reactivity against eight non-target *Phytophthora* species. The detection limit was 1 femtogram of *P. agathidicida* DNA via endpoint PCR. Performance assessment against 65 soil samples from kauri forests revealed *P. agathidicida* in 69% of samples compared to only 11% detected by existing methods. By eliminating the need for baiting, our assay enhances the speed, accuracy, and accessibility of testing, thereby facilitating more comprehensive monitoring and improved disease management.

**IMPORTANCE:**

*Phytophthora* species are notorious plant pathogens responsible for severe dieback and root rot diseases, significantly impacting crops, forests, and irreplaceable natural ecosystems. Rapid and accurate detection of these pathogens is essential for effective disease management. In New Zealand, *P. agathidicida* threatens the country’s endemic kauri forests. In this study, we developed and validated a PCR-based method for detecting *P. agathidicida* oospores in soil. Oospores are long-lived, thick-walled spores that serve as key propagules for survival in soil and the spread of disease. Their robust structure and dormant state make them particularly challenging to detect using traditional soil baiting techniques or DNA-based methods. Our method is fast, accurate, and requires minimal equipment, enabling local testing and thereby empowering communities and enhancing surveillance efforts. Although developed for *P. agathidicida*, this method could be adapted for other plant pathogens, potentially improving disease management across various agricultural and ecological contexts.

## INTRODUCTION

*Phytophthora* species are among the most destructive plant pathogens globally, causing significant damage to crops, forests, and natural ecosystems (1–7). Early and accurate detection of these pathogens is crucial for implementing effective disease management strategies and containing outbreaks.

*Phytophthora agathidicida* is the causative agent of kauri dieback disease, which poses a significant threat to New Zealand’s iconic kauri forests (8). *P. agathidicida* is thought to be an introduced pathogen and has been spreading throughout the kauri forests of New Zealand since, at least, the 1970s, when its symptoms were first reported (9). This soil-borne pathogen infects kauri trees through their roots, leading to bleeding lesions on the trunk, collar rot, defoliation at the crown, and, ultimately, tree death (10, 11). Human activities, including the movement of contaminated soils on shoes, equipment, and nursery stock, have been implicated in the spread of *P. agathidicida* (8). However, the pathogen can be present in an area for several years before the onset of visible disease symptoms (8), hindering containment and management efforts. This long latency period (1–10 years), coupled with the need to limit the spread of *P. agathidicida*, necessitates methods that detect pathogen presence rather than relying on visible symptoms.

Soil baiting is a widely employed technique for detecting *Phytophthora* species, with various baiting methods used by land managers and regulatory agencies worldwide to identify and monitor potentially harmful *Phytophthora* species (1, 12–15), including *P. agathidicida* (16). This method involves introducing susceptible plant materials as bait into flooded soil samples, stimulating the germination and growth of potential *Phytophthora* spores. If produced, motile zoospores infect the baits, allowing for pathogen identification through subsequent culturing or molecular detection methods. However, soil baiting has several limitations: it is time-consuming, is labor-intensive, and can fail to detect slow-growing or fastidious species that are outcompeted by faster-growing organisms during the baiting or culturing process (12, 17–20). Furthermore, *Phytophthora* species such as *P. agathidicida*, which produce resting spores (e.g., oospores), can be difficult to detect if the spores remain dormant (21); soil baiting for *P. agathidicida* has been shown to have a high false-negative rate (22, 23). DNA-based methods, such as loop-mediated isothermal amplification (LAMP), have been integrated with traditional baiting techniques (i.e., a hybrid baiting-LAMP assay) to reduce the overall processing time and/or improve sensitivity (24). However, the other limitations of traditional baiting remain.

An alternative is directly testing soil for the presence of *Phytophthora* DNA using molecular methods such as PCR, LAMP, or metabarcoding approaches (20, 21, 25). However, these techniques face several challenges, including limitations with soil sample volume (25, 26), ineffective oospore lysis (27), and co-extraction of PCR inhibitors (28, 29). The effectiveness of baiting-based methods compared with DNA methods can be variable: although metabarcoding typically identifies a greater overall species diversity (20, 21, 30–32), in some cases, it fails to detect *Phytophthora* species that can be successfully isolated via baiting, or it yields fewer detections for a target species (20, 33). For example, a recent study on the *Phytophthora* communities associated with kauri found a substantially lower detection rate for *P. agathidicida* using metabarcoding compared with baiting (52% versus 94% of total *P. agathidicida* detections, respectively [33]). Given these limitations, there is a clear need for improved techniques to reliably and efficiently detect target *Phytophthora* species in soil.

This study presents the development and validation of a method for detecting *P. agathidicida* oospores in soil using PCR. The optimized method includes a technique to separate oospores from bulk soil, improved oospore lysis and DNA extraction, and a specific primer pair/probe for PCR amplification of *P. agathidicida* DNA. The performance of this method was assessed against a panel of 65 soil samples from kauri forests that had been previously tested using the currently approved hybrid soil baiting-LAMP approach (16, 24). Although our method has been developed specifically for *P. agathidicida*, the principles underlying the method could be applied to other *Phytophthora* species and/or other spore-forming pathogens, potentially contributing to improved disease management strategies across various agricultural and ecological contexts.

## MATERIALS AND METHODS

### Identification of target sequences and *in silico* analyses of specificity and primer design

Potential repetitive sequences to target were identified in the genome sequence of *P. agathidicida* isolate 3770 (GenBank Assembly: GCA_025722995.1) (34) using the Repeat Finder plugin of Geneious 2023.0.4. The most abundant repeat was an 8,636 bp sequence predicted to encode a long terminal repeat (LTR) retrotransposon of the Copia family.

The presence and specificity of the identified LTR retrotransposon within the *Phytophthora* genus were investigated using comparative genomics. Reads from multiple *P. agathidicida* isolates (Sequence Read Archive (SRA) project ID: SRP158337) and other *Phytophthora* species commonly present in New Zealand forests (*Phytophthora pluvialis, Phytophthora cinnamomi, Phytophthora multivora, Phytophthora kernoviae, Phytophthora* taxon Totara; SRA project ID: SRP061438) (35) were aligned to the transposon sequence using Bowtie 2 v2.4.4 (36). The resulting alignments were analyzed using SAMtools v1.13 (37) to identify regions specific to *P. agathidicida* (i.e., no reads from other species align) versus those shared among *Phytophthora* species. Although a small region in the reverse transcriptase gene showed similarities between *Phytophthora* species, the LTR region identified with the Repeat Finder plugin was unique to *P. agathidicida*. To further assess specificity, BLAST searches were performed against the NCBI nucleotide (nt) database for both the entire transposon and the LTR region. Similar analyses were conducted for previously used target regions (ITS and *cob*).

To compare the copy number of the LTR region with previously used target regions (26, 38) (ITS and *cob*), reads from *P. agathidicida* isolate 3770 (34) (SRA ID: SRR14752241) were mapped to these sequences using Geneious Read Mapper at the highest sensitivity setting. The copy number of each region was estimated by dividing its read depth by the average read depth of the genome (excluding mitochondrial sequences).

After confirming the LTR region as a specific target *in silico*, Primer3 v4.1.0 (39) was used to design an optimal primer pair within this region. This resulted in the following primers: PA-LTR-for: 5ʹ-ACGCGCTCTGTTTCTTTAGC-3ʹ and PA-LTR-rev: 5ʹ-GCGGGCTTCCATTCAATTCA-3ʹ.

### *In vitro* tests of primer specificity and sensitivity using endpoint PCR

To assess primer specificity, genomic DNA from three isolates of *P. agathidicida* (isolates NZFS 3770, 3772, and 3813) and eight other *Phytophthora* species (*P. castaneae*, *P. heveae*, *P. cocois*, *P. cinnamomi*, *P. cryptogea*, *P. kernoviae, P. multivora*, *P. pseudocryptogea*; Table S1) was extracted to be used as input in endpoint PCR assays. To obtain the genomic DNA of each, the isolates were cultured as mycelial mats in potato-dextrose broth (Difco) until they covered approximately 50% of the Petri dish surface. The harvested mats were washed with sterile water, dried using Kimwipes, and ground to a fine powder in liquid nitrogen using a mortar and pestle. Genomic DNA was extracted using the DNeasy Plant Mini kit (Qiagen) following the manufacturer’s protocol. DNA concentrations were quantified using the Qubit dsDNA High Sensitivity Assay Kit and NanoPhotometer NP80 (Implen).

One nanogram (ng) of genomic DNA from each *Phytophthora* species was used in PCR assays with our PA-LTR primer set. The previously reported PTA_ITS_F2 (5ʹ-AACCAATAGTTGGGGGCGA-3ʹ) and PTA_ITS_R3 (5ʹ-CTCGCCATGATAGAGCTCGTC-3ʹ) primers (22, 26, 40) were also tested with *P. agathidicida* and the other three Clade 5 species for comparison. Primer sensitivity was evaluated using 10-fold dilutions of *P. agathidicida* NZFS 3770 genomic DNA (100 pg to 0.001 pg) with both our PA-LTR and ITS primers. The endpoint PCR assays were performed in 20 µL reactions using Quick-Load Taq 2X Master Mix (New England BioLabs) with a final primer concentration of 500 nM. All PCR reactions were performed using a T100 Thermal Cycler (Bio-Rad). PCR thermal cycling conditions for the PA-LTR primer pair were: 95°C for 30 s, 35–40 cycles of 95°C for 22 s, 55°C for 30 s, and 68°C for 7 s, and a final extension of 68°C for 5 min. For the ITS Clade 5 primer pair (PTA_ITS_F2 and PTA_ITS_R3), the thermal cycling conditions were as reported previously (40): 95°C for 60 s, 35–40 cycles of 95°C for 30 s, 57°C for 30 s, and 68°C for 30 s, and final extension of 68°C for 5 min. PCR products were analyzed by gel electrophoresis on a 2% (wt/vol) agarose gel pre-stained with GelGreen Nucleic Acid Gel Stain.

### Soil panel used for method optimization

A panel of 10 soil samples with known PA status was used for our method development. The samples had been previously collected, air-dried, and tested using a hybrid baiting-LAMP assay (24) by BioSense Limited as part of routine kauri dieback monitoring. The panel of soil samples included six positive and four negative samples from properties across Auckland and Northland regions. The DNA from each soil sample was ultimately extracted using three different techniques (described in more detail below): (i) extraction of 250 mg of soil using the DNeasy PowerSoil Pro kit (Qiagen) performed according to the manufacturer’s instructions, (ii) extraction of 250 mg of soil using the DNeasy PowerSoil Pro kit (Qiagen) but with SK38 Soil Grinding Beads (2 mL; Precellys) instead of the supplied PowerBead Pro Tubes, and (iii) ~12.5 g of soil reduced to approximately 250 mg using the double-filter bag method protocol before extraction with the DNeasy PowerSoil Pro kit and SK38 Soil Grinding Beads. All three methods were followed by a DNeasy PowerClean Pro Cleanup Kit, with a final elution volume of 100 µL. The extracted DNA was then used as input in endpoint PCRs using the PA-LTR primers and cycling conditions described above. For each sample, genomic DNA was spiked into a second PCR reaction to detect potential inhibition; any inhibition control samples with no band or a weak band in these spiked reactions were classified as inhibited.

### Comparison of oospore lysis methods and DNA extraction efficiency

Purified *P. agathidicida* oospores were subjected to different lysis methods to compare lysis efficiency. To obtain oospores, *P. agathidicida* (NZFS 3770) mycelial mats were cultured for 6–8 weeks, followed by homogeniation of the mats and filtration to remove mycelial debris, as described previously (41). The resulting suspensions of purified oospores were stored in 5 mL of sterile water at 4°C in the dark until use. Oospore concentrations were estimated using one of two methods: either by counting 10 µL samples in triplicate with a hemocytometer to calculate an average or once with a Scepter 3.0 Handheld Automated Cell Counter (Merck) using a 60 µm sensor, with oospores diluted in 1× phosphate-buffered saline and 0.001% Tween.

Approximately 280,000 cultured *P. agathidicida* oospores were used for each lysis trial. Lysis was performed using a 10 min bead-beating step with a Vortex-Genie 2 Vortex fitted with a horizontal adapter (Qiagen). Either PowerBead Pro tubes or SK38 Soil Grinding Beads (2 mL; Bertin Corp) were used, both performed in triplicate. Lysis was observed at 10× magnification using a light microscope (Olympus CKX53 microscope).

### Method for isolation of oospores from the soil via double-filter bags

A double-filter bag method was developed to isolate oospores from soil, removing both larger soil debris and smaller particulates (42). For each sample, a 6 × 6 cm pouch made from 90 µm nylon membrane was filled with soil until approximately two-thirds full. Although the soil samples are prepared volumetrically, the mass per sample was recorded (mean sample weight 12.5 g ± 3 g SD). This pouch was then sealed and enclosed within a 7.5 × 8 cm pouch constructed from a 25 µm nylon membrane. Each double-filter bag was submerged in a 50 mL tube filled with distilled water, vortexed, and allowed to soak for 30 min. Following this initial soaking, the water was discarded and replaced with fresh distilled water, after which the pouch was soaked for an additional 15 min. The outer pouch was then removed and cut open, whereas the inner pouch was discarded. The outer pouch was rinsed with 25 mL of distilled water to create a soil slurry. This slurry was subsequently centrifuged at 3,000 × *g* for 2 min to pellet the solids. The supernatant was discarded, and the resulting pellet served as the starting material for subsequent DNA extraction using the PowerSoil Pro kit with SK38 bead beating tubes, followed by the PowerClean Pro Cleanup Kit and PCR/qPCR using the PA-LTR primers.

### Assessment of the combined method on a larger panel of field samples

The combined and optimized DNA extraction method was applied to 65 field samples. These samples had been previously collected, air-dried, and tested using a hybrid baiting-LAMP assay (24) by Biosense Limited as part of routine kauri dieback monitoring. Of the 65 samples, 7 (11%) of the provided samples were positive, and 58 were negative (89%) using the hybrid baiting-LAMP assay. In addition to the hybrid baiting-LAMP assay results, we were also provided with observational data collected during field sampling. This data included observations such as the presence/absence of *P. agathidicida*-related symptoms such as lesions. To minimize potential researcher bias, the identity and previous data for the individual samples were blinded to the researchers performing the DNA extractions and PCR assays, that is, the researchers conducting the experiments (OC or ED) were only provided with sample numbers and no other data.

The 65 samples were extracted using the optimized method (i.e. double-bag filtration of oospores, followed by DNA extraction using SK38 bead tubes). The extracted DNA samples were then subjected to three assays: First, standard endpoint PCR was conducted using the PA-LTR primers with 20 µL reactions, using 1 or 3 µL of the sample DNA input and supplemented with PCR Enhancer Cocktail P (PEC-P, DNA Polymerase Technology). To assess the potential inhibition, an identical PCR was conducted per DNA sample, with the addition of 1 pg of *P. agathidicida* genomic DNA. Second, all 65 DNA samples were also subjected to endpoint-nested PCR with ITS primers designed for *Phytophthora* (ITS6 and 5.8 S-1R) (30) and Clade 5 (PTA_ITS_F2 and PTA_ITS_R3) (22, 26). These reactions were 20 µL, with 1 or 3 µL of the sample input for the first PCR and 2 µL for the second, both supplemented with PEC-P. The varied inputs for the endpoint PCRs accounted for potential inhibition and low pathogen levels.

### Quantitative PCR (qPCR) assays

A TaqMan probe, named PA-LTR-TaqMan probe, was designed for the center of the sequence to adapt the PA-LTR primer set for qPCR use: 5′-FAM-TAGTATGCGCTTTTGAGGAAGCGTAA-BHQ1-3′. For all qPCR assays, fluorescein amidite (FAM; Macrogen) was used as the fluorescent dye, and black hole quencher 1 (BHQ1; Macrogen) was used as the quencher. Thermal cycling was performed using a QuantStudio3 Real-Time PCR System (Applied Biosystems) with the following settings: 95°C for 2 min, followed by 45 cycles of 95°C for 5 s, 60°C for 30 s. Reactions were conducted in 10 µL reactions using Quantabio PerfeCTa qPCR ToughMix (2X), final primer concentration of 500 nM, probe concentration of 250 nM, and 1 µL of input DNA. For the standard curve, genomic DNA isolated from *P. agathidicida* isolate 3770 was used, and the initial concentration was assessed using the Qubit dsDNA High Sensitivity Assay Kit. For the standard curve, 10 concentrations (ranging from 0.01 fg to 10,000,000 fg [10 ng]) were prepared using DNA LoBind tubes (Eppendorf), with nine replicates per concentration measured. A standard curve was generated by plotting the log_10_ DNA concentrations against the cycle threshold (CT) values; this standard curve was used to calculate the DNA concentration of *P. agathidicida* in the soil samples. For soil samples, 10 µL qPCRs were performed in triplicate for each sample, using the conditions described above but supplemented with PEC-P to account for potential inhibition. Negative controls (distilled water only) were included in each run.

## RESULTS

### Primer design and *in silico* validation

Our bioinformatics analysis identified a highly repetitive transposon sequence in the *P. agathidicida* genome. This sequence, approximately 8,500 bp long, is present more than 100 times throughout the genome and in each chromosome (Fig. S1A); no reads from other tested *Phytophthora* genomes mapped to this sequence. The sequence belongs to the LTR/Copia transposon family, flanked by a ~500 bp long terminal repeat (LTR) region on both sides (Fig. S1B).

Further BLAST analysis confirmed the high specificity of this region to *P. agathidicida*, with the LTR region returning hits only from *P. agathidicida* (Tables S2 and S3). This specificity contrasts with primer sequence targets from previous molecular *P. agathidicida* detection studies (24, 26). For example, the region amplified by ITS_F2 and R3 matches exactly with sequences from many other Clade 5 *Phytophthora* species (Supplementary Data: Hit Table ITS). Similarly, the mitochondrial *cob* gene shows high similarity (96% nucleotide identity) to other Clade 5 species (Supplementary Data: Hit Table *cob*), rendering this region only suitable in combination with LAMP, which uses six primers in total to improve specificity.

We utilized raw reads from previous *P. agathidicida* sequencing projects (34) to estimate the copy number of our target region compared with these previous targets (Table 1). This copy number estimation indicated that the LTR sequence has a read depth 470 times higher than the genome average. In comparison, the ITS sequence is estimated to occur 217 times per genome copy, whereas the mitochondrial *cob* sequence has an estimated copy number of 105 (Table 1).

**TABLE 1:** Comparison of LTR primers versus primers used in previous studies

Study	Target	Specificity	Copy number	Locus	PCR method
Than et al.	ITS	Clade 5 *Phytophthora* sp.	217	Chromosome 10	PCR/qPCR
Winkworth et al.	*cob*	*P. agathidicida*	105	Mitochondrial	LAMP
This study	LTR	*P. agathidicida*	470	Chromosomes 1–10	PCR/qPCR

The read-depth analysis across the transposon revealed that LTR sequences are four times more abundant than any other part of the transposon (Fig. S1). Combined, these results suggested that the LTR region has high abundance and conservation within *P. agathidicida* isolates and, based on the currently available sequence data, has no significant similarity with other *Phytophthora* species, including closely related Clade 5 species.

### *In vitro* tests of primer specificity and sensitivity using endpoint PCR

**Fig 1:**
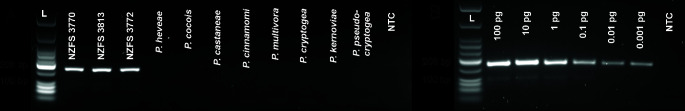
Specificity and sensitivity of the PA-LTR primers using PCR. (A) Specificity assessment using 1 ng of genomic DNA from various *Phytophthora* species. *P. agathidicida* isolates NZFS 3770, 3813, and 3772 each produced the expected ~160 bp PCR product, whereas the other *Phytophthora* species showed no amplification. (B) Sensitivity assessment using *P. agathidicida* NZFS 3770 DNA concentrations from 100 pg to 0.001 pg. PCR products were detected down to 0.001 pg (1 fg) input DNA. For (A) and (B), the PCR products were resolved on 2% TAE agarose gels stained with GelGreen Nucleic Acid Gel Stain. L: NEB low molecular weight ladder; NTC: No template control.

We designed the primer set PA-LTR primers to target a repeat region of the *P. agathidicida* genome*,* with a predicted product size of ~160 bp. To assess primer specificity, three geographically distinct genetic groups of *P. agathidicida* (isolates NZFS 3770, 3772, and 3813) were selected for this study based on previous genetic characterization (38). Additionally, eight other *Phytophthora* species were included for comparison. These species comprised Clade 5 representatives (*P. castaneae*, *P. heveae*, and *P. cocois*) and species commonly found in New Zealand forests (*P. cinnamomi*, *P. cryptogea*, *P. kernoviae, P. multivora*, and *P. pseudocryptogea*) (33, 35). The primers successfully amplified the target product in all three *P. agathidicida* isolates tested, whereas no amplification was observed for any other *Phytophthora* species, demonstrating their specificity for *P. agathidicida* (Fig. 1A). This represents improved performance compared with the PTA_ITS primers, which amplify other Clade 5 species (Fig. S3A) (26).

The PA-LTR primers also exhibited high sensitivity, successfully detecting the 0.001 pg of *P. agathidicida* genomic DNA after 40 PCR cycles (Fig. 1B). Given that the full genome of *P. agathidicida* is 57 Mb (34), with an estimated diploid genome weight of 125 fg (Table S4), this suggests that the primer set can detect amounts below one genome equivalent. There is a secondary product observed at 98/103 bp at the higher concentrations of *P. agathidicida* DNA tested (Fig. 1B). This secondary product is attributed to deletions in chromosome 6 of the genome.

We also compared the sensitivity of PA-LTR primers with the previously designed Clade 5-specific ITS primers (22, 26, 40) using a serial dilution of *P. agathidicida* DNA. The PA-LTR primers demonstrated a 10-fold lower detection limit than the ITS primers (0.001 pg vs 0.01 pg; Fig. 1B vs (Fig. S3B). This improved performance is likely due to the higher copy number of the target sequence within the *P. agathidicida* genome (Table 1). Overall, the PA-LTR primers demonstrated improved specificity for *P. agathidicida* and a 10-fold increase in sensitivity using PCR compared with the previously used ITS primers.

### Optimization of oospore lysis and DNA extraction

Next, the PA-LTR primer pair was evaluated against a panel of 10 field-collected soil samples with known PA status. DNA was extracted from these samples using the DNeasy PowerSoil Pro Kit, using 250 mg of soil and the standard manufacturer’s protocol, followed by the DNeasy PowerClean Pro Cleanup Kit. For each sample, genomic DNA was spiked into a second PCR reaction to detect potential inhibition (inhibition control; IC). Using this method and the PA-LTR primer pair in PCR reactions, *P. agathidicida* was detected in only two of the six known positive samples (Samples 3 and 5), and one sample (Sample 2) was inhibited (Fig. S4). Overall, the detection rate at this stage was only 33% (two of six known positives detected). Given the high sensitivity of the PA-LTR primer pair, we suspected that the false-negative results might be due to insufficient oospore lysis. To investigate this, the lysis of purified oospores was assessed using microscopy.

**Fig 2:**
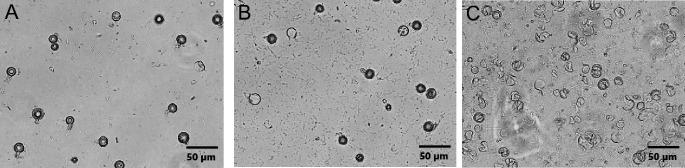
Comparison of oospore lysis efficiency using different bead tubes. (A) Purified oospores pre-lysis. (B) Oospores after 10 minutes of lysis using PowerBead pro tubes. (C) Oospores after 10 minutes of lysis using SK38 bead tubes.

As shown in Fig. 2, the use of PowerBead Pro tubes results in poor oospore lysis, with the majority of oospores remaining intact after 10 minutes of vortexing (Fig. 2A: oospores pre-lysis; Fig. 2B oospores post-lysis with PowerBead Pro tubes; Fig. S5 additional images). We then tested SK38 Soil Grinding Beads using the same lysis procedure. The SK38 bead tubes achieved nearly complete oospore lysis (Fig. 2C and (Fig. S6).

Following the observation that SK38 Soil Grinding Beads significantly improved oospore lysis, we re-tested the same panel of 10 soil samples. Again, we extracted DNA from 250 mg of each soil sample using the DNeasy PowerSoil Pro Kit but substituting the supplied PowerBead Pro tubes with SK38 bead tubes. The use of SK38 bead tubes improved our detection rate to 50%, with three of the six known positive samples successfully detected using endpoint PCR; inbition of some samples was still observed (Fig. S7). Overall, substituting the SK38 bead beating tubes improved the detection of *P. agathidicida* in soil samples compared with the standard lysis method using PowerBead Pro tubes. However, the detection rate remained worse than the existing hybrid baiting-LAMP assay; hence, further optimization was needed.

### Combined method for oospore isolation, DNA extraction, and endpoint PCR

Given the potentially low density of oospores in field soil samples, we next aimed to increase the sample volume beyond 250 mg. For context, the hybrid baiting-LAMP assay typically uses 250–500 g of soil (16, 24), which vastly exceeds the input capacity of the PowerSoil Pro kit (and most other commercial DNA extraction kits). Instead, we employed a size-based separation method to isolate and concentrate the oospores from the soil. This method utilized two filter bags with different pore sizes (90 µm and 25 µm) to trap oospores between the two layers, thereby removing larger debris (>90 µm, e.g., coarse soil) and smaller particles (<25 µm, e.g., silt, bacteria). Following this passive filtration step, the oospores and residual soil (~250 mg) were collected, pelleted, and subjected to DNA extraction using our optimized extraction method (i.e., DNeasy PowerSoil Pro Kit with SK38 Soil Grinding Bead Tubes). The extracted oospore DNA (oDNA) was then analyzed using an endpoint PCR assay with the PA-LTR primers.

**Fig 3:**
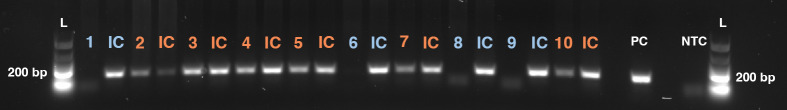
Performance of the combined oDNA extraction and endpoint PCR method on soil samples with known PA status. The soil sample panel consists of six PA-positive samples (Samples 2, 3, 4, 5, 7, and 10), indicated in orange, and four PA-negative samples (Samples 1, 6, 8, and 9), indicated in blue. Approximately 5–15 g of each soil sample was extracted using the optimized method, and endpoint PCRs were performed using the PA-LTR primers and 1 µL of extracted DNA. Inhibition control (IC) PCR reactions were conducted for each sample by spiking a second PCR reaction with 1 pg of PA genomic DNA. PCR products of the expected size (~160 bp) were detected in all six PA-positive samples (2, 3, 4, 5, 7, and 10). No PCR products were observed in the four PA-negative samples (1, 6, 8, and 9). The inhibition controls yielded PCR products for all samples, suggesting no substantial PCR inhibition in any sample. The positive control (PC; 1 pg PA genomic DNA) and no template control (NTC) performed as expected. The PCR products were resolved on 2% TAE agarose gels stained with GelGreen Nucleic Acid Gel Stain. L: NEB low molecular weight ladder.

This combined method was evaluated using the panel of 10 soil samples with known PA status. As shown in Fig. 3, the combined method yielded PCR products of the expected size (~160 bp) for all six *P. agathidicida*-positive (PA-positive samples: 2, 3, 4, 5, 7, and 10) and none of the *P. agathidicida*-negative samples (PA-negative samples: 1, 6, 8, and 9). Notably, PCR inhibition was reduced in Sample 2, resulting in a positive detection for the first time in our assays. Overall, the combined method successfully detected 100% of the positive samples, with no false-positive or false-negative PCR results observed in the 10-sample panel.

### Performance on a wider data set of 65 previously characterized field samples

**Fig 4:**
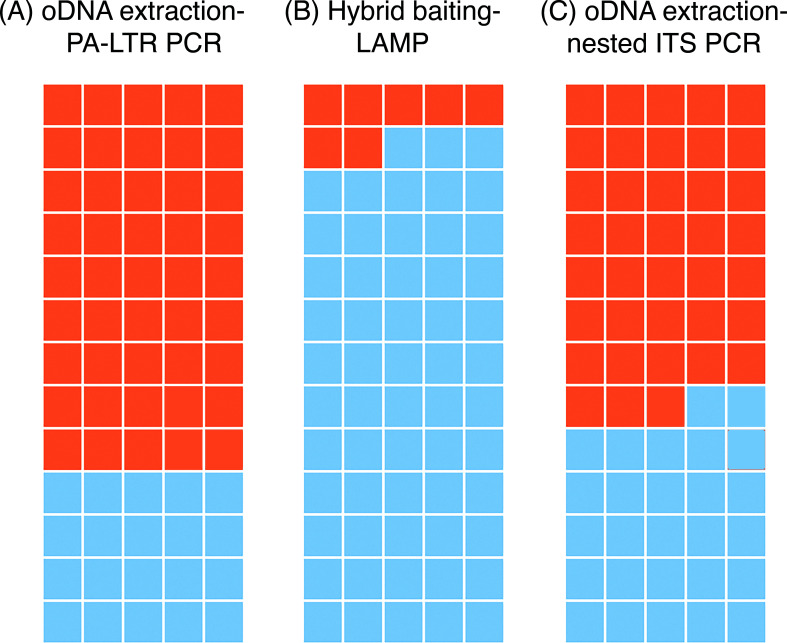
Comparison of *P. agathidicida* detections from 65 field-collected soil samples. (A) Endpoint PCR results using the PA-LTR primer pair on extracted oospore DNA (oDNA). (B) Previous results were obtained using the hybrid baiting-LAMP assay. (C) Nested PCR results using the Clade 5-specific ITS primer set on extracted oDNA. Each square represents an individual soil sample, with colors indicating the detection status: orange for PA-positive samples and blue for PA-negative samples.

The combined and optimized protocol was applied to a larger panel of 65 previously tested soil samples from the Northland and Auckland regions. Using our optimized oDNA extraction method with endpoint PCR, 45 samples (69%) yielded a clear PCR product of the expected size and were classified as PA-positive, whereas 20 samples (31%) were PCR-negative (Fig. 4A). These results were then compared with the previous hybrid baiting-LAMP assay results (Fig. 4B). All 20 negative oDNA samples tested by endpoint PCR were also negative in the hybrid-LAMP assay. However, our method exhibited a significantly higher detection rate compared with the hybrid-LAMP assay. Specifically, 38 samples were positive in our assay but negative in the hybrid-LAMP assay (Fig. 4A and B).

To further validate these findings, all 65 extracted oDNA samples were cross-checked using nested PCR (Fig. 4C) and a different primer set. The nested PCR employed *Phytophthora* spp. universal primers (ITS6 and 5.8 S-1R) were used as outer primers in the first round, and the PTA_ITS_F2 and PTA_ITS_R3 primers were used in the second amplification round to amplify *P. agathidicida*. Although the second-round primers also amplify other Clade 5 species in addition to *P. agathidicida*, the primer pair has been the most specific available and, therefore, has been widely used to detect and/or quantify *P. agathidicida* DNA (33, 43–45). Of the 45 samples classified as PA-positive using endpoint PCR, 38 were also positive in the nested PCRs, providing additional evidence for the presence of *P. agathidicida* DNA in these samples. The 20 samples that were negative using the PA-LTR primer pair (and the hybrid baiting-LAMP assay) were also negative using nested PCR. Seven samples had differing classifications as PA-positive versus negative using the two different PCR methods. Overall, 58 of 65 samples were consistently classified as positive or negative for *P. agathidicida* using our method for oDNA extraction from soil when tested by two independent PCR assays.

Next, we performed qPCR to explore whether there was a relationship between the observed detection status using these different methods and the amount of *P. agathidicida* DNA present in the soil samples. A standard curve was generated using *P. agathidicida* genomic DNA (Fig. S8). Using the PA-LTR primers and the PA-LTR-probe, qPCR exhibited a primer efficiency of 96%. The limit of detection (LOD) was determined to be 0.4 fg of *P. agathidicida* genomic DNA, corresponding to a cycle threshold (CT) of 37.8. The limit of quantification (LOQ) was found to be 29 fg, corresponding to a CT of 31.5.

**Fig 5:**
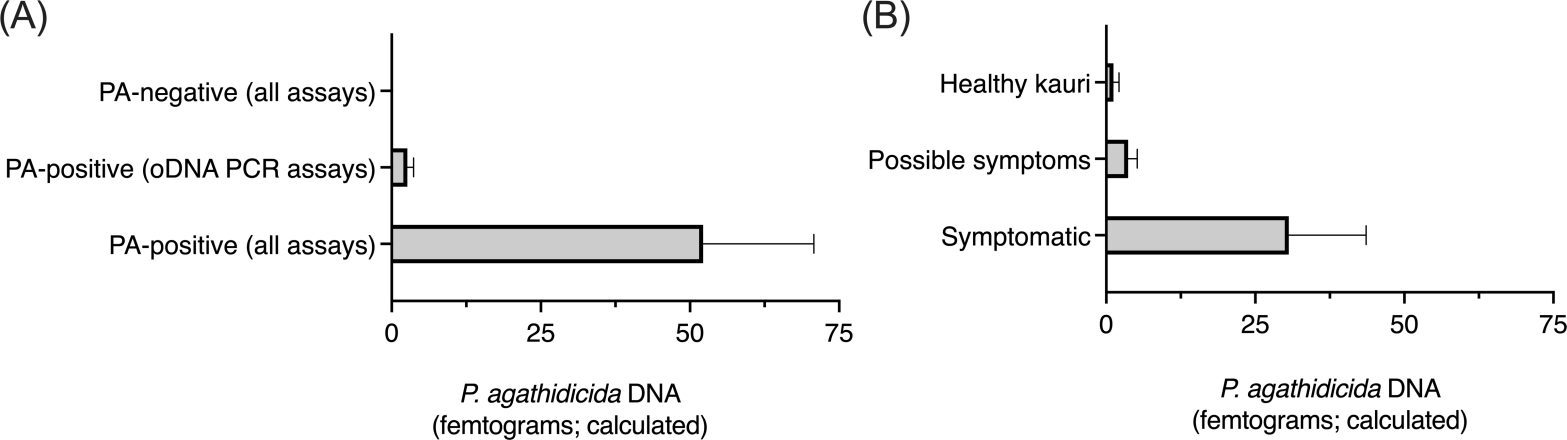
Calculated *P. agathidicida* DNA quantity for different conditions, determined via qPCR. (A) The average calculated the amount of *P. agathidicida* DNA was classified by the method of detection. Bars represent the mean, and error bars indicate the standard error of the mean for each category: PA-negative (all assays), *n* = 20; PA-positive (one or both of the oDNA PCR assays), *n* = 38; PA-positive (all assays, including oDNA PCRs and hybrid baiting-LAMP), *n* = 7. (B) Calculated amount of *P. agathidicida* DNA in each observed health status of the sampled kauri. Bars represent the mean, and error bars indicate the standard error of the mean for each category: healthy kauri, *n* = 31; kauri with possible dieback symptoms, *n* = 22; kauri with dieback symptoms, *n* = 12.

As shown in Fig. 5A, the samples classified as PA-negative across all assay methods had a calculated average of 0 fg *P. agathidicida* DNA. In contrast, samples classified as PA-positive across all assay methods, including the hybrid baiting-LAMP assay, had, on average, 52 fg of *P. agathidicida* DNA. Samples that tested positive using the oDNA-PCR assays but were not detected by the hybrid baiting-LAMP assay had lower average amounts of *P. agathidicida* DNA present, averaging 2.6 fg. Although this average is above the calculated qPCR limit of detection (LOD) of 0.4 fg, it is below the limit of quantification (LOQ; 29 fg); thus, this value should be interpreted as an estimate rather than a precise quantification.

We also examined how the amount of *P. agathidicida* DNA varied across kauri trees with different reported symptom statuses, including healthy kauri, kauri with possible symptoms of infection, and kauri with visible symptoms of *P. agathidicida* infection. As shown in Fig. 5B, the average amount of *P. agathidicida* DNA in soil samples from healthy kauri was 1.2 fg. In contrast, soil samples from trees with possible symptoms of *P. agathidicida* infection had a slightly higher average of 3.7 fg. The highest average amount of *P. agathidicida* DNA was found in soil samples from kauri with visible symptoms of *P. agathidicida* infection, with an average of 31 fg.

## DISCUSSION

**Fig 6:**
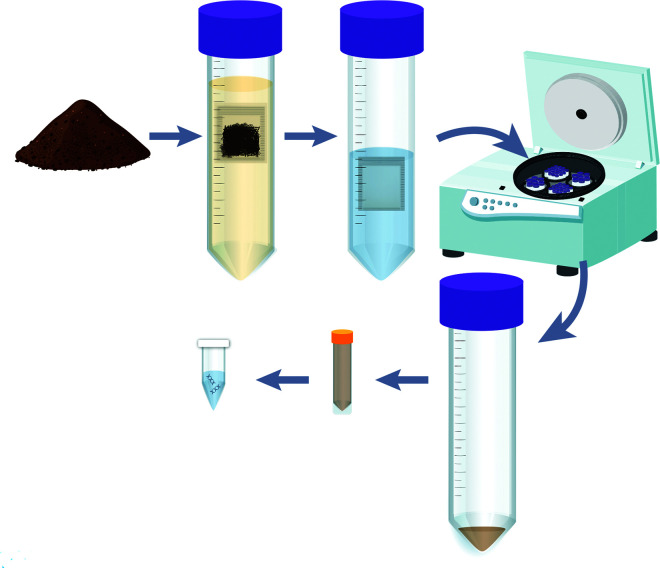
Overview of the optimized method for extracting oospore DNA from soil samples for molecular analysis. First, a 6 × 6 cm pouch made from 90 µm nylon membrane is filled with soil until approximately two-thirds full (typically ~12 g of soil). This bag is sealed and placed within a 7.5 × 8 cm pouch constructed from a 25 µm nylon membrane. The outer bag filter is also sealed, and then, the double-filter bag is soaked in water for a minimum of 30 min. During this time, oospores separate by diffusion from the small and large fractions of the bulk soil, remaining in the outer filter bag. The water and inner filter bag (containing bulk soil and other large particulates) are discarded, and the outer bag is subsequently rinsed and centrifuged, resulting in a pellet that contains oospores, other similarly sized particles, and residual soil. This pellet undergoes an optimized DNA extraction process using SK38 bead tubes, followed by PCR amplification using the PA-LTR primer pair or other DNA-based methods. This method enables the efficient isolation and molecular analysis of oospores from soil samples. Image adapted with permission from Reference (42).

This study reports the development and validation of a novel DNA extraction method and PCR assay for reliable detection of *P. agathidicida* in soil samples. The method, illustrated in Fig. 6, first isolates oospores from soil based on size, using a double-layer filter bag. This approach effectively reduces the initial soil sample to approximately 250 mg, which is then subjected to an optimized DNA extraction protocol. Our extraction method incorporates SK38 Soil Grinding beads, replacing the standard beads supplied with the PowerSoil Pro extraction kit. This modification significantly enhances cell lysis efficiency, resulting in improved DNA extraction. The extracted oospore DNA is then analyzed using endpoint PCR assay with primers designed to target a highly repetitive region of DNA specific to the *P. agathidicida* genome.

Our method for isolating oospores from soil effectively addresses many of the limitations associated with direct soil testing, particularly low pathogen titers, soil heterogeneity, and the small sample volumes usable with most DNA extraction kits (24, 33). Although other approaches, such as sieving-centrifugation with sucrose solutions, have been successfully used to separate spores of various organisms from bulk soil for detection and/or quantification (46–48), our approach using filter bags offers several advantages: it is faster, simpler, and reduces the risk of cross-contamination associated with sieving. Although we focused on *P. agathidicida* oospores, this method has the potential for broader applications. By adjusting the pore size of the filter bags, it can be adapted to isolate various pathogens or organisms of interest (e.g., fungal spores and nematodes) from soil samples. The size of the filter bags can also be customized; for example, larger bags can be used when the pathogen load is expected to be low, and a greater soil volume needs to be processed.

Our study also underscores the critical role of oospore lysis in successful DNA extraction from soil. Oospores possess robust cell walls that are resistant to common DNA extraction protocols (27). This resistance can lead to intact oospores after the lysis step, resulting in inaccessible DNA for amplification. Consequently, this may cause underestimation or false negatives in molecular detection assays. We observed this phenomenon when using the PowerSoil Pro kit, where most oospores remained intact after lysis, rendering known *P. agathidicida*-positive samples undetectable by PCR. These findings underscore the importance of verifying oospore lysis before employing DNA-based detection methods, particularly when these methods are used to report pathogen distribution or inform disease management strategies. Our optimized extraction uses SK38 Soil Grinding beads instead of the supplied beads from the PowerSoil Pro extraction kit, which results in improved cell lysis and, therefore, DNA extraction. Although the extracted oospore DNA can be used as the input in various DNA-based detection methods (e.g., LAMP, qPCR, and DNA metabarcoding), in this study, we have focused on validating an easy-to-implement endpoint PCR assay.

The endpoint PCR assay developed and validated in this study offers several advantages over existing methods for detecting *P. agathidicida*. It consistently detected as little as 1 fg of *P. agathidicida* DNA while not amplifying closely related species. This is improved specificity compared with the primers described by Than et al. (26), which, although commonly used to detect or quantify *P. agathidicida* in soil (33, 43–45), can also cross-amplify other Clade 5 *Phytophthora* species. Combined with our optimized extraction process, we achieved a significantly higher detection rate than the hybrid baiting-LAMP assay. In a panel of 65 soil samples, our assay detected *P. agathidicida* in 45 samples (69%), compared with only seven samples (11%) detected by the hybrid baiting-LAMP assay. A nested PCR using an independent primer set had similar results to the LTR primer pair (38/65 positive; 58%), highlighting that our oDNA extraction method yields comparable results even when used with other primer pairs/PCR methods. Although semi-quantitative, the qPCR results also provided valuable insights into the detection limits for field samples using these different methods.

Our method also offers practical benefits compared with the currently approved methods for *P. agathidicida* detection (16, 49), which rely on soil baiting followed by either LAMP (8) or culturing and morphological assessment (50). The dependence on soil baiting is problematic for several reasons. First, it is a time-consuming process, requiring at least 10 days (16), with additional time needed for downstream molecular or laboratory culture tests. Second, baiting requires larger soil samples (~250 g) than our method (~10–15 g), increasing collection, transportation, and disposal challenges. Finally, due to the classification of *P. agathidicida* as an “unwanted organism” by the New Zealand government, soil baiting can only be conducted in approved containment facilities or laboratories, limiting testing accessibility and increasing costs. These issues of cost and accessibility are particularly significant due to the cultural importance of kauri, especially for indigenous Māori: kauri are considered sacred, living ancestors whose health and survival are intertwined with the well-being of the people and the land (51). Iwi (tribes) in kauri regions have responsibility for these forests and, in many areas, lead the disease surveillance and management efforts. However, the need for approved containment facilities when using soil baiting means samples must be sent away to specialized laboratories for testing.

Our endpoint PCR method offers a viable alternative to traditional laboratory-based approaches, enabling on-site pathogen detection. By eliminating the need for baiting and culturing, our method removes the requirement for a containment facility. The extraction and PCR-based assay can be performed using compact, portable equipment such as a Bento Lab, which integrates a thermal cycler, powered electrophoresis system, blue light transilluminator, and centrifuge into a single unit. The integrated extraction and PCR protocol can be easily scaled to process multiple samples in parallel, and samples can be processed from start to finish in a single day. This capability significantly enhances both the speed and cost-effectiveness of testing.

The ability to conduct local testing with minimal equipment requirements represents a significant advancement that can empower local communities and amplify surveillance efforts. This capability will facilitate more comprehensive monitoring of kauri forests, supporting strategic protection decisions that can help protect kauri forest health.

## SUPPLEMENTAL MATERIAL

Supplemental material spectrum.00135-25-s0001.pdf Fig. S1 to S8; Tables S1 to S4.

ASM does not own the copyrights to Supplemental Material that may be linked to, or accessed through, an article. The authors have granted ASM a non-exclusive, world-wide license to publish the Supplemental Material files. Please contact the corresponding author directly for reuse.

## References

[1] Erwin DC, Ribeiro OK. 1996. Phytophthora diseases worldwide. The American Phytopathological Society (APS Press).

[2] Kamoun S, Furzer O, Jones JDG, Judelson HS, Ali GS, Dalio RJD, Roy SG, Schena L, Zambounis A, Panabières F, et al.. 2015. The top 10 oomycete pathogens in molecular plant pathology. Mol Plant Pathol 16:413–434. doi:10.1111/mpp.12190 25178392 PMC6638381

[3] Scott P, Bader MKF, Burgess T, Hardy G, Williams N. 2019. Global biogeography and invasion risk of the plant pathogen genus Phytophthora. Environ Sci Policy 101:175–182. doi:10.1016/j.envsci.2019.08.020

[4] Fones HN, Bebber DP, Chaloner TM, Kay WT, Steinberg G, Gurr SJ. 2020. Threats to global food security from emerging fungal and oomycete crop pathogens. Nat Food 1:332–342. doi:10.1038/s43016-020-0075-0 37128085

[5] Green S, Cooke DEL, Dunn M, Barwell L, Purse B, Chapman DS, Valatin G, Schlenzig A, Barbrook J, Pettitt T, Price C, Pérez-Sierra A, Frederickson-Matika D, Pritchard L, Thorpe P, Cock PJA, Randall E, Keillor B, Marzano M. 2021. PHYTO-THREATS: Addressing threats to UK forests and woodlands from Phytophthora; identifying risks of spread in trade and methods for mitigation. Forests 12:1617. doi:10.3390/f12121617

[6] Brasier C, Scanu B, Cooke D, Jung T. 2022*.* Phytophthora: an ancient, historic, biologically and structurally cohesive and evolutionarily successful generic concept in need of preservation. IMA Fungus 13:12. doi:10.1186/s43008-022-00097-z 35761420 PMC9235178

[7] Bose T, Spies CFJ, Hammerbacher A, Coutinho TA. 2023. Phytophthora: an underestimated threat to agriculture, forestry, and natural ecosystems in sub-Saharan Africa. Mycol Progress 22:78. doi:10.1007/s11557-023-01926-0

[8] Bradshaw RE, Bellgard SE, Black A, Burns BR, Gerth ML, McDougal RL, Scott PM, Waipara NW, Weir BS, Williams NM, Winkworth RC, Ashcroft T, Bradley EL, Dijkwel PP, Guo Y, Lacey RF, Mesarich CH, Panda P, Horner IJ. 2020. Phytophthora agathidicida: research progress, cultural perspectives and knowledge gaps in the control and management of kauri dieback in New Zealand . Plant Pathol 69:3–16. doi:10.1111/ppa.13104

[9] Gadgil PD. 1974. Phytophthora heveae, a pathogen of kauri. NZ J For Sci:459–463.

[10] Beever RE, Waipara NW, Ramsfield TD, Dick MA, Horner IJ. 2009. Kauri (Agathis australis) Under Threat From Phytophthora? Phytophthoras in Forests and Natural Ecosystems. doi:10.2737/PSW-GTR-221

[11] Weir BS, Paderes EP, Anand N, Uchida JY, Pennycock SR, Bellgard SE, Beever RE. 2015. A taxonomic revision of Phytophthora Clade 5 including two new species Phytophthora agathidicida and P. cocois. Phytotaxa 205:21–38. doi:10.11646/phytotaxa.205.1.2

[12] O’Brien PA, Williams N, Hardy GES. 2009. Detecting Phytophthora. Crit Rev Microbiol 35:169–181. doi:10.1080/10408410902831518 19624253

[13] Martin FN, Abad ZG, Balci Y, Ivors K. 2012. Identification and detection of Phytophthora: reviewing our progress, identifying our needs. Plant Dis 96:1080–1103. doi:10.1094/PDIS-12-11-1036-FE 30727075

[14] Burgess TI, López‐Villamor A, Paap T, Williams B, Belhaj R, Crone M, Dunstan W, Howard K, Hardy GEStJ. 2021. Towards a best practice methodology for the detection of Phytophthora species in soils . Plant Pathol 70:604–614. doi:10.1111/ppa.13312

[15] Pérez-Sierra A, Jung MH, Jung T. 2022. Survey and monitoring of Phytophthora species in natural ecosystems: methods for sampling, isolation, purification, storage, and pathogenicity tests. Methods Mol Biol 2536:13–49. doi:10.1007/978-1-0716-2517-0_2 35819596

[16] Tiakina Kauri Standard Operating Procedure. 2023. Approved soil baiting method for Phytophthora agathidicida. Ministry for Primary Industries, Biosecurity New Zealand.Version 1.2.

[17] Ferguson AJ, Jeffers SN. 1999. Detecting multiple species of Phytophthora in container mixes from ornamental crop nurseries. Plant Dis 83:1129–1136. doi:10.1094/PDIS.1999.83.12.1129 30841137

[18] Sarker SR, McComb J, Burgess TI. 2020. Antimicrobials in Phytophthora isolation media and the growth of Phytophthora species. Plant Pathol 69:1426–1436. doi:10.1111/ppa.13224

[19] Sarker SR, McComb J, Burgess TI, Hardy GESJ. 2021. Timing and abundance of sporangia production and zoospore release influences the recovery of different Phytophthora species by baiting. Fungal Biol 125:477–484. doi:10.1016/j.funbio.2021.01.009 34024595

[20] Sarker SR, Burgess TI, Hardy GESJ, McComb J. 2023. Closing the gap between the number of Phytophthora species isolated through baiting a soil sample and the number revealed through metabarcoding. Mycol Progress 22:39. doi:10.1007/s11557-023-01892-7

[21] Bačová A, Cooke DEL, Milenković I, Májek T, Nagy ZÁ, Corcobado T, Randall E, Keillor B, Cock PJA, Jung MH, Jung T, Tomšovský M. 2024. Hidden Phytophthora diversity unveiled in tree nurseries of the Czech Republic with traditional and metabarcoding techniques. Eur J Plant Pathol 170:131–156. doi:10.1007/s10658-024-02886-1

[22] McDougal R, Bellgard S, Scott P, Ganley B. 2014. Comparison of a real-time PCR assay and a soil bioassay technique for detection of Phytophthora taxon Agathis from soil, ID 53789

[23] Singh J, Curran-Cournane F, Waipara N, Schwendenmann L, Lear G. 2017. Comparison of methods used to detect the organism responsible for kauri dieback, Phytophthora agathidicida, from soil samples. Auckland Council Technical Report 2017/019

[24] Winkworth RC, Nelson BCW, Bellgard SE, Probst CM, McLenachan PA, Lockhart PJ. 2020. A LAMP at the end of the tunnel: A rapid, field deployable assay for the kauri dieback pathogen, Phytophthora agathidicida. PLoS One 15:e0224007. doi:10.1371/journal.pone.0224007 31978166 PMC6980612

[25] Elliot M, Schlenzig A, Harris CM, Meagher TR, Green S. 2015. An improved method for qPCR detection of three Phytophthora spp. in forest and woodland soils in northern Britain. For Pathol 45:537–539. doi:10.1111/efp.12224

[26] Than DJ, Hughes KJD, Boonhan N, Tomlinson JA, Woodhall JW, Bellgard SE. 2013. A TaqMan real-time PCR assay for the detection of Phytophthora “taxon Agathis” in soil, pathogen of Kauri in New Zealand. For Pathol 43:324–330. doi:10.1111/efp.1203

[27] Lees AK, Sullivan L, Lynott JS, Cullen DW. 2012. Development of a quantitative real‐time PCR assay for Phytophthora infestans and its applicability to leaf, tuber and soil samples . Plant Pathol 61:867–876. doi:10.1111/j.1365-3059.2011.02574.x

[28] Hargreaves SK, Roberto AA, Hofmockel KS. 2013. Reaction- and sample-specific inhibition affect standardization of qPCR assays of soil bacterial communities. Soil Biol and Biochem 59:89–97. doi:10.1016/j.soilbio.2013.01.007

[29] Wang H, Qi J, Xiao D, Wang Z, Tian K. 2017. A re-evaluation of dilution for eliminating PCR inhibition in soil DNA samples. Soil Biol Biochem 106:109–118. doi:10.1016/j.soilbio.2016.12.011

[30] Scibetta S, Schena L, Chimento A, Cacciola SO, Cooke DEL. 2012. A molecular method to assess Phytophthora diversity in environmental samples. J Microbiol Methods 88:356–368. doi:10.1016/j.mimet.2011.12.012 22226752

[31] La Spada F, Cock PJA, Randall E, Pane A, Cooke DEL, Cacciola SO. 2022. DNA Metabarcoding and Isolation by baiting complement each other in revealing Phytophthora diversity in anthropized and natural ecosystems. JoF 8:330. doi:10.3390/jof8040330 35448560 PMC9028584

[32] Sarker SR, McComb J, Hardy GESJ, Burgess TI. 2023. Sample volume affects the number of Phytophthora and Phytopythium species detected by soil baiting. Eur J Plant Pathol 166:303–313. doi:10.1007/s10658-023-02661-8

[33] Hunter S, Horner I, Hosking J, Carroll E, Newland J, Arnet M, Waipara N, Burns B, Scott P, Williams N. 2024. Phytophthora communities associated with Agathis australis (kauri) in Te Wao Nui o Tiriwa/Waitākere Ranges, New Zealand. Forests 15:735. doi:10.3390/f15050735

[34] Cox MP, Guo Y, Winter DJ, Sen D, Cauldron NC, Shiller J, Bradley EL, Ganley AR, Gerth ML, Lacey RF, McDougal RL, Panda P, Williams NM, Grunwald NJ, Mesarich CH, Bradshaw RE. 2022. Chromosome-level assembly of the Phytophthora agathidicida genome reveals adaptation in effector gene families. Front Microbiol 13:1038444. doi:10.3389/fmicb.2022.1038444 36406440 PMC9667082

[35] Studholme DJ, McDougal RL, Sambles C, Hansen E, Hardy G, Grant M, Ganley RJ, Williams NM. 2016. Genome sequences of six Phytophthora species associated with forests in New Zealand. Genom Data 7:54–56. doi:10.1016/j.gdata.2015.11.015 26981359 PMC4778589

[36] Langmead B, Salzberg SL. 2012. Fast gapped-read alignment with Bowtie 2. Nat Methods 9:357–359. doi:10.1038/nmeth.1923 22388286 PMC3322381

[37] Danecek P, Bonfield JK, Liddle J, Marshall J, Ohan V, Pollard MO, Whitwham A, Keane T, McCarthy SA, Davies RM, Li H. 2021. Twelve years of SAMtools and BCFtools. Gigascience 10:giab008. doi:10.1093/gigascience/giab008 33590861 PMC7931819

[38] Winkworth RC, Bellgard SE, McLenachan PA, Lockhart PJ. 2021. The mitogenome of Phytophthora agathidicida: Evidence for a not so recent arrival of the “kauri killing” Phytophthora in New Zealand. PLoS One 16:e0250422. doi:10.1371/journal.pone.0250422 34019564 PMC8139493

[39] Untergasser A, Cutcutache I, Koressaar T, Ye J, Faircloth BC, Remm M, Rozen SG. 2012. Primer3—new capabilities and interfaces. Nucleic Acids Res 40:e115–e115. doi:10.1093/nar/gks596 22730293 PMC3424584

[40] Thurston AM. 2021. Detection and prevention: Improving techniques to manage Phytophthora agathidicida, the causal agent of kauri dieback. Master of Science. Lincoln University.

[41] Lacey RF, Fairhurst MJ, Daley KJ, Ngata-Aerengamate TA, Patterson HR, Patrick WM, Gerth ML. 2021. Assessing the effectiveness of oxathiapiprolin toward Phytophthora agathidicida, the causal agent of kauri dieback disease. FEMS Microbes 2:xtab016. doi:10.1093/femsmc/xtab016 37334227 PMC10117877

[42] Palmer JTT, Gerth ML. 2025. A method for the separation of Phytophthora oospores from soil for DNA-based detection. Methods Mol Biol 2892:139–149. doi:10.1007/978-1-0716-4330-3_10 39729274

[43] Schwendenmann L, Michalzik B. 2019. Dissolved and particulate carbon and nitrogen fluxes along a Phytophthora agathidicida infection gradient in a kauri (Agathis australis) dominated forest. Fungal Ecol 42:100861. doi:10.1016/j.funeco.2019.08.005

[44] Schwendenmann L, Michalzik B. 2021. Impact of Phytophthora agathidicida infection on canopy and forest floor plant nutrient concentrations and fluxes in a kauri-dominated forest. Ecol Evol 11:4310–4324. doi:10.1002/ece3.7326 33976812 PMC8093678

[45] Hunter S, Waipara N, Burns B, Scott P, Williams N. 2024. Impacts of phosphite treatment on Phytophthora community assemblages and inoculum abundances in Phytophthora-infected forest soil. Trees, Forests and People 18:100687. doi:10.1016/j.tfp.2024.100687

[46] Wang PH, Chang CW. 2003. Detection of the low-germination-rate resting oospores of Pythium myriotylum from soil by PCR. Lett Appl Microbiol 36:157–161. doi:10.1046/j.1472-765x.2003.01287.x 12581375

[47] Pavón CF, Babadoost M, Lambert KN. 2008. Quantification of Phytophthora capsici oospores in soil by sieving-centrifugation and real-time polymerase chain reaction. Plant Dis 92:143–149. doi:10.1094/PDIS-92-1-0143 30786362

[48] Nishimura F, Fujisawa H, Mori M, Nakashima C, Nakanishi M, Iwamoto Y, Mimuro G, Nakamura Y, Komori M, Ikeda K. 2022. Monitoring of Peronospora destructor oospores from field samples using real-time PCR. Plant Pathol 71:1784–1792. doi:10.1111/ppa.13604

[49] Tiakina Kauri Standard Operating Procedure. 2023. Approved soil and root sampling method for Phytophthora agathidicida detection Biosecurity New Zealand, Ministry for Primary Industries. Version 1.0.

[50] Beever RE, Bellgard SE, Dick MA, Horner IJ, Ramsfield TD. 2010. Detection of Phytophthora taxon Agathis (PTA). Landcare Report LC0910/137. Prepared for the Ministry for Agriculture & Forestry, Biosecurity New Zealand (on behalf of the Kauri Dieback Joint Agency).

[51] Pomare P, Tassell-Matamua N, Lindsay N, Masters-Awatere B, Dell K, Erueti B, Te Rangi M. 2023. Te mauri o te kauri me te ngahere: indigenous knowledge, te taiao (the Environment) and wellbeing. Knowl Cult 11:55–83. doi:10.22381/kc11120234

