# Supplementary material for: Improved isolation and PCR detection of Phytophthora agathidicida oospores from soils
Source: Microbiol Spectr. 2025 Apr 8;13(5):e00135-25. doi: 10.1128/spectrum.00135-25 (PMC12073851; doi:10.1128/spectrum.00135-25)
Supplement: Supplemental material — Fig. S1 to S8; Tables S1 to S4. [file spectrum.00135-25-s0001.pdf]

Supplementary Information

Improved isolation and PCR detection of *Phytophthora agathidicida* oospores from soils

Jade T. T. Palmer, Jochem N. A. Vink, Leticia M. Castro, Oliver J. S. Craig, Emily E. Davison and Monica L. Gerth*

*Corresponding Author: M.L. Gerth; Email: monica.gerth@vuw.ac.nz

Supplementary Figures	Pages 1 - 8
Supplementary Tables	Pages 9 – 14
References	Page 15

Supplementary Figures

Supplementary Figure 1

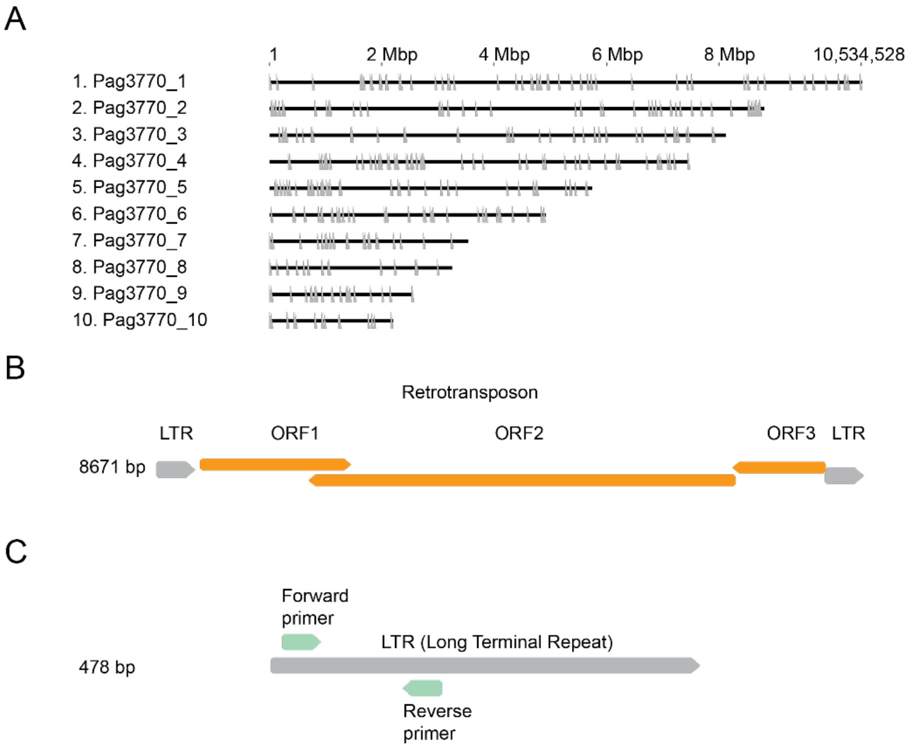

Supplementary Figure 1. The target repeat region in the *P. agathidicida* genome. A) Abundance of target retrotransposon throughout *P. agathidicida* genome. B) Schematic of the retrotransposon region, including the flanking Long Terminal Repeat (LTR) regions.(C) Schematic of the PA-LTR primer binding sites within the LTR region.

Supplementary Figure 2

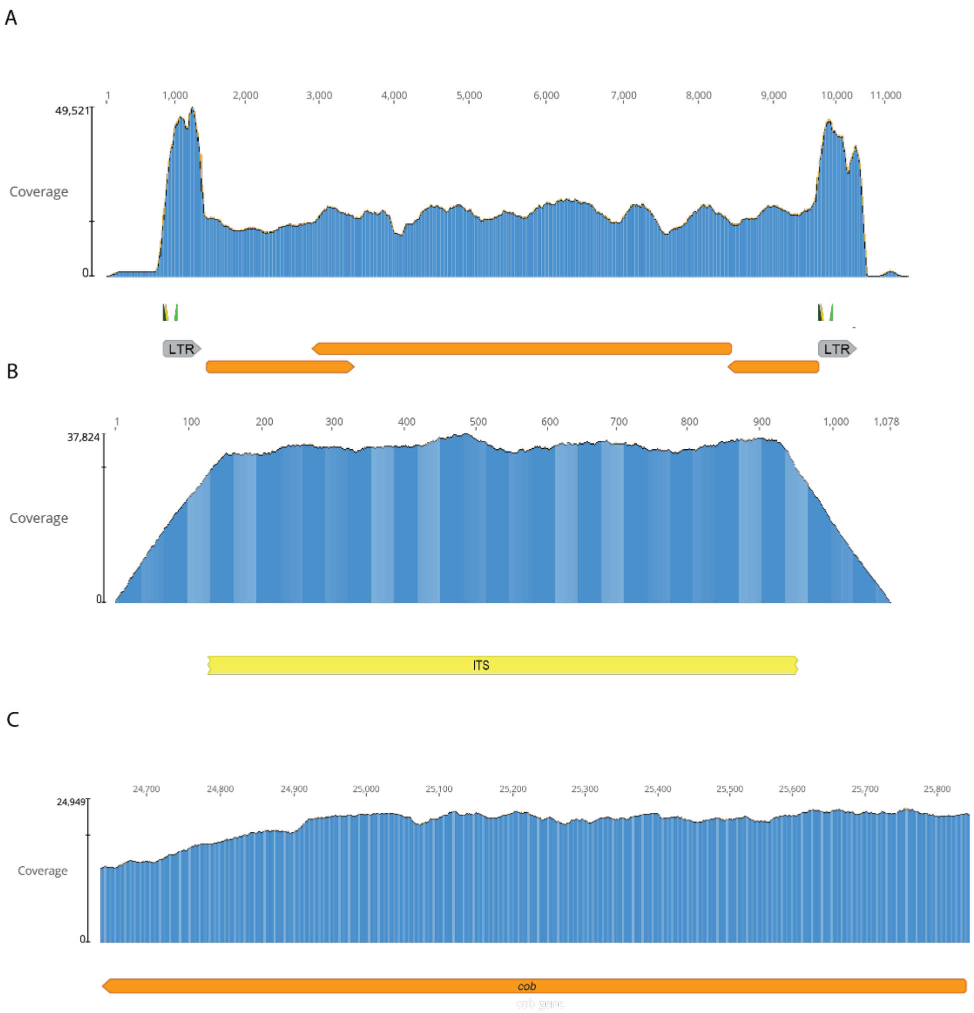

Supplementary Figure 2 Coverage of reads (SRR14752241) from *P. agathidicida* isolate 3770 (Cox et al., 2022). (A) Mapping to the transposon region that was targeted in this study. B) Mapping to the ITS region (Than et al., 2013) . (C) Mapping to the *cob* region (Winkworth, 2020). ORFs are indicated in orange, the long terminal repeat (LTR) region in grey and the ITS region in yellow. The average coverage within the targeted region was used to estimate the genome copy number of each target.

Supplementary Figure 3

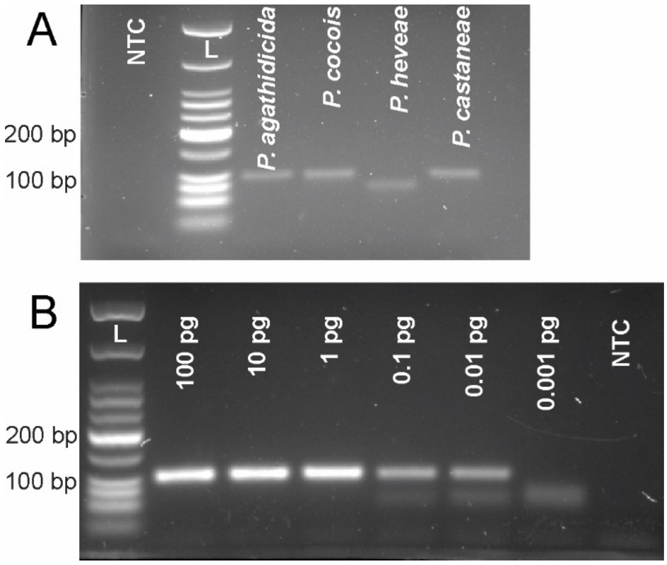

Supplementary Figure 3. PCR amplification of clade 5 *Phytophthora* species with the PTA_ITS_F2 and PTA_ITS_R3 primers (McDougal et al., 2014; Than et al., 2013). (A) Products were produced with all clade 5 *Phytophthora* species tested, demonstrating that the primers are not specific for *P. agathidicida*. (B) PCR products were detectable down to 0.01 pg of *P. agathidicida* input DNA; 0.001 pg of input DNA did not produce a visible PCR product. For panels (A) and (B) PCR products were resolved on 2% TAE agarose gels stained with GelGreen Nucleic Acid Gel Stain. NTC: No template control; L: NEB low molecular weight ladder.

Supplementary Figure 4.

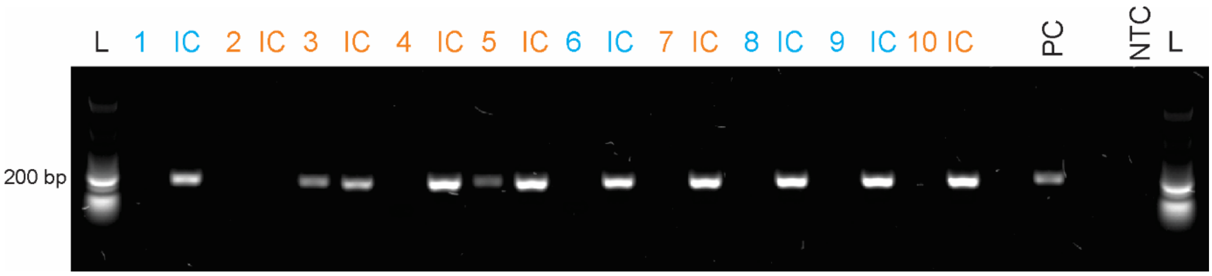

Supplementary Figure 4. Performance of the PA-TE primers in PCR reactions using DNA extracted via the standard DNeasy PowerSoil Pro kit. The soil samples extracted were a panel of six PA-positive samples (Samples 2, 3, 4, 5, 7, and 10), indicated in orange, and four PA-negative samples (Samples 1, 6, 8, and 9), indicated in blue. Approximately 250 mg of each soil sample was extracted using the PowerSoil Pro kit, and end-point PCRs were performed using the PA-TE primers and 1 µL of extracted DNA. Inhibition control (IC) PCR reactions were conducted for each sample by spiking a second PCR reaction with 1 ng of PA genomic DNA. PCR products of the expected size (~160 bp) were detected in only two (Samples 3 and 5) of the six PA-positive samples. The PCR products were resolved on 2% TAE agarose gels stained with GelGreen Nucleic Acid Gel Stain. L: NEB low molecular weight ladder; IC: Spiked inhibition controls. PC: Positive control; NTC: No template control.

Supplementary Figure 5.

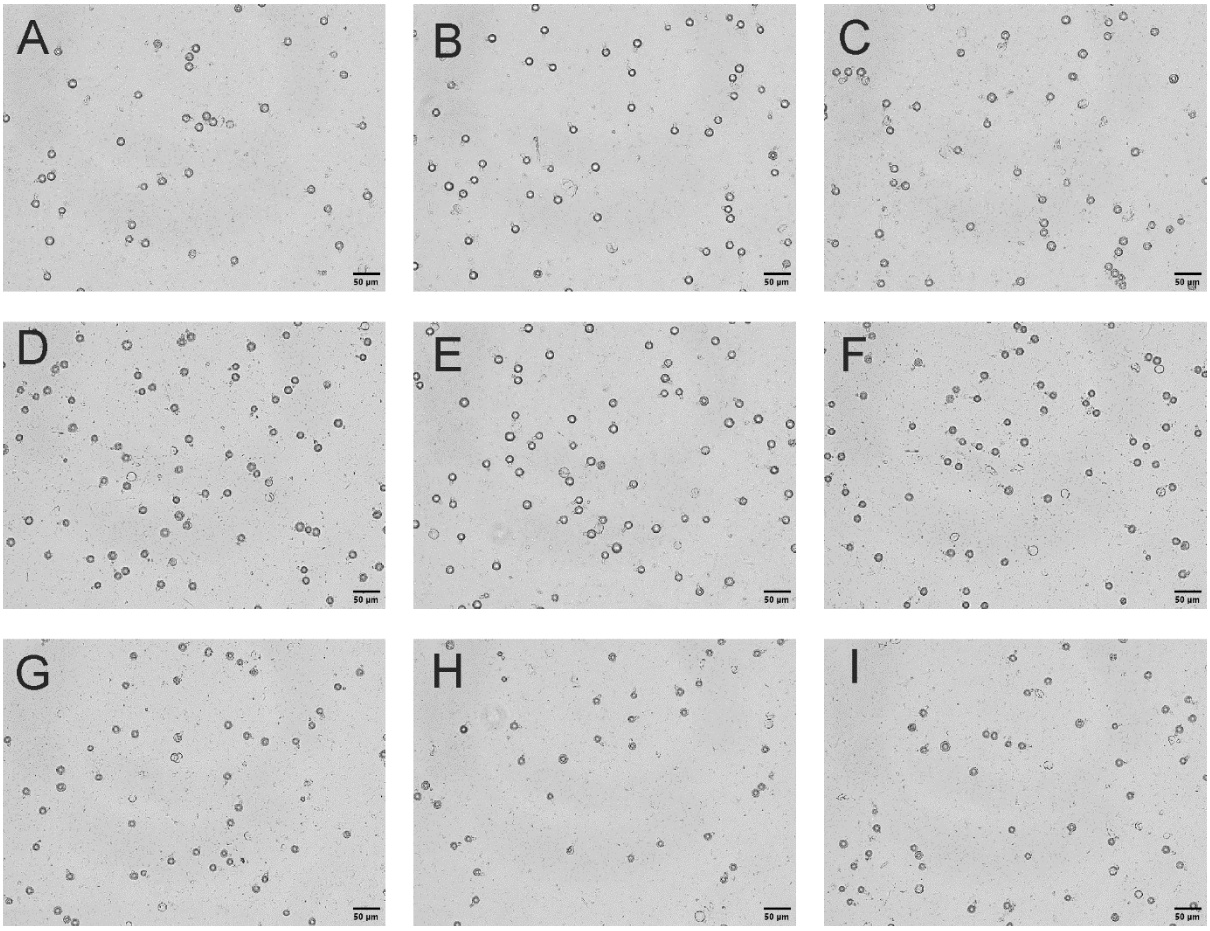

Supplementary Figure 5. Microscopy images of oospores after lysis with supplied PowerBead Pro tubes. Three oospore samples were subjected to 10 minutes of vortex bead-beating, and three fields of view were captured per sample. Panels A-C: Sample 1. Panels D-F: Sample 2. Panels G-I: Sample 3. Scale bars represent 50 µm.

Supplementary Figure 6.

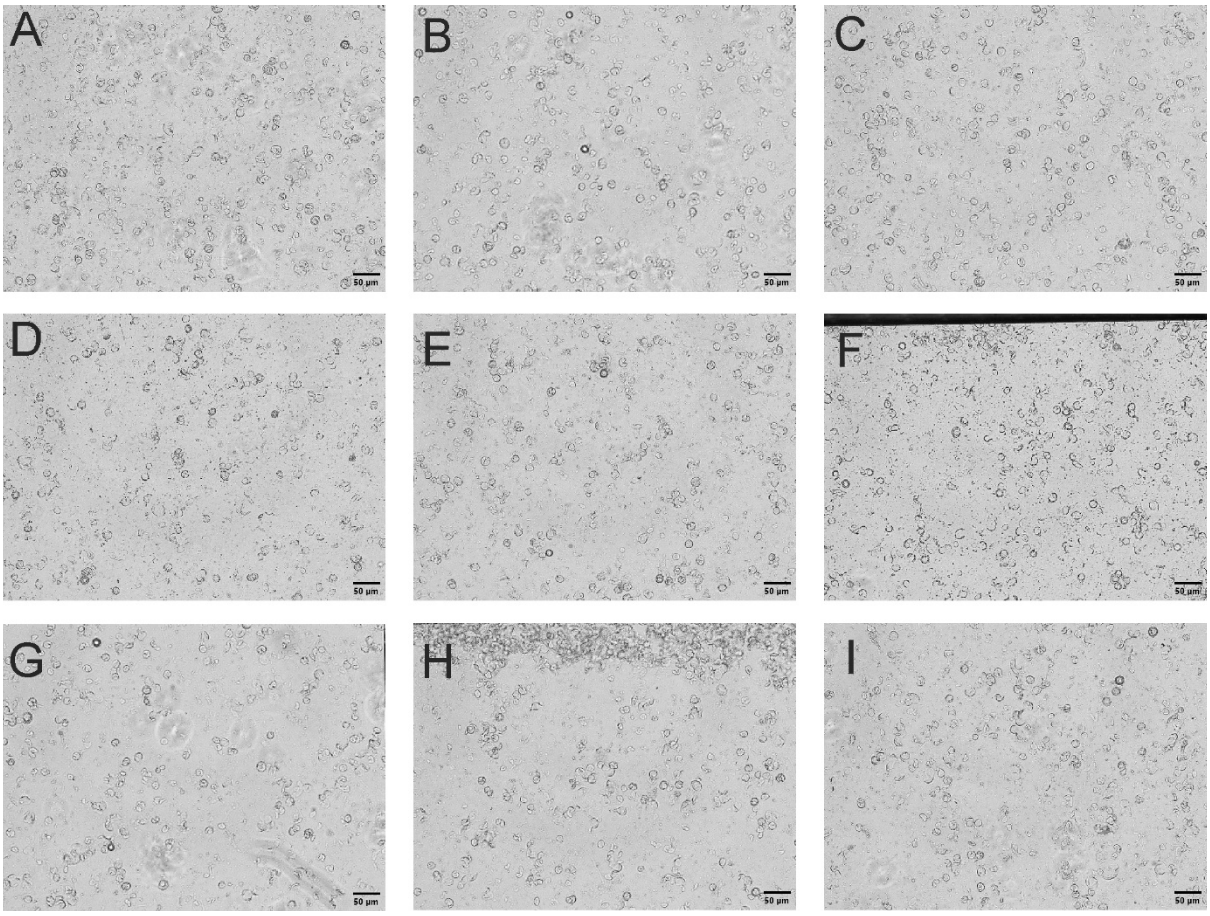

Supplementary Figure 6. Microscopy images of oospores after lysis with SK38 soil grinding beads. Three oospore samples were subjected to 10 minutes of vortex bead-beating, and three fields of view were captured per sample. Panels A-C: Sample 1. Panels D-F: Sample 2. Panels G-I: Sample 3. Scale bars represent 50 μm.

Supplementary Figure 7

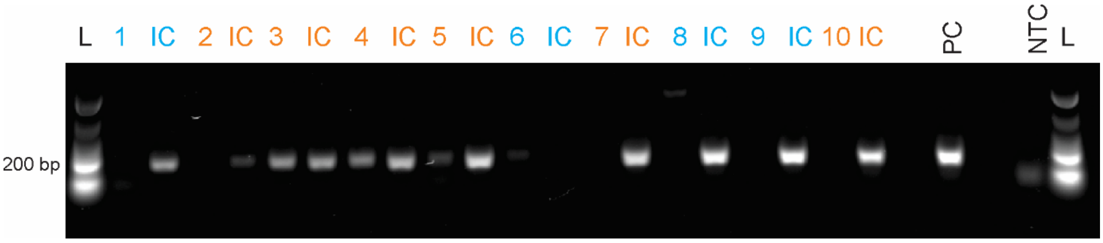

Supplementary Figure 7. Performance of the PA-TE primers in PCR reactions using DNA extracted via the DNaeasy PowerSoil Pro kit with SK38 Soil Grinding Beads The soil samples extracted were a panel of six PA-positive samples (Samples 2, 3, 4, 5, 7, and 10), indicated in orange, and four PA-negative samples (Samples 1, 6, 8, and 9), indicated in blue. Approximately 250 mg of each soil sample was extracted using the PowerSoil Pro kit with SK38 beads, and end-point PCRs were performed using the PA-TE primers and 1 µL of extracted DNA. Inhibition control (IC) PCR reactions were conducted for each sample by spiking a second PCR reaction with 1 ng of PA genomic DNA. PCR products of the expected size (~160 bp) were detected in three (Samples 3, 4 and 5) of the six PA-positive samples. A fourth sample (#6) was deemed inconclusive, as the corresponding inhibition control failed. The PCR products were resolved on 2% TAE agarose gels stained with GelGreen Nucleic Acid Gel Stain. L: NEB low molecular weight ladder; #IC: Spiked inhibition controls. PC: Positive control; NTC: No template control.

Supplementary Figure 8

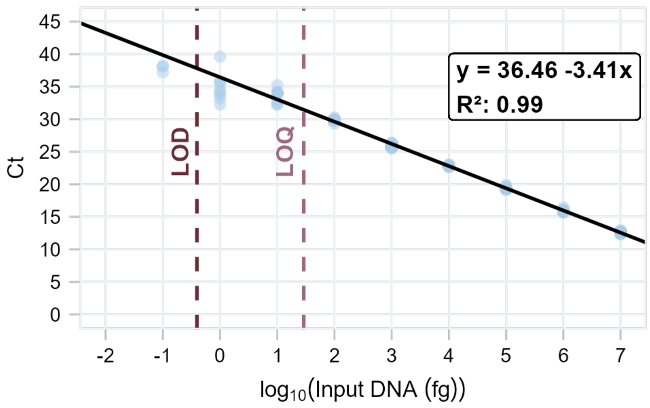

Supplementary Figure 8. qPCR standard curve of *P. agathidicida* genomic DNA. Ten concentrations (ranging from 0.01 fg to 10,000,000 fg (10 ng) were measured using nine replicates per concentration. The lowest concentration (0.01 fg) was not detected in all replicates and, therefore, not shown. The slope, intersect and R^2 of the trendline were calculated from the six highest concentrations. The limit of detection (LOD, dark red) and limit of quantification (LOQ, light red) were calculated according to previously developed algorithms (Klymus et al., 2020). Using the PA-LTR primers and the PA-LTR-probe, qPCR exhibited a primer efficiency of 96%. The limit of detection (LOD) was determined to be 0.4 fg of *P. agathidicida* genomic DNA, corresponding to a cycle threshold (CT) of 37.8. The limit of quantification (LOQ) was calculated to be 29 fg, corresponding to a CT of 31.5.

Supplementary Tables

Supplementary Table 1. List of *Phytophthora* species and isolates used for primer testing.

Isolate	Clade	Obtained from
P. agathidicida 3770	5	Scion
P. agathidicida 3772	5	Scion
P. agathidicida 3813	5	Scion
P. cinnamomi NZFS 3910	7	Scion
P. heveae ICMP 16691	5	Landcare Research
P. cocois ICMP 16948	5	Landcare Research
P. castaneae ICMP 19434	5	Landcare Research
P. multivora ICMP 21656	2	Landcare Research
P. cryptogea PDD 26147	8	Landcare Research
P. kernoviae ICMP 15082	10	Landcare Research
P. pseudocryptogea ICMP 21674	8	Landcare Research

Supplementary Table 2. LTR Hit table. Hits from BLAST search (blastn; nr/nt) of LTR sequence.

Description	Scientific Name	Max Score	Total Score	Query Cover	E value	% Identity	Acc. Len	Accession
Phytophthora agathidicida strain 3770 chromosome 8	*Phytophthora agathidicida*	863	19146	100%	0	100	3225653	CP106978.1
Phytophthora agathidicida strain 3770 chromosome 7	*Phytophthora agathidicida*	863	27949	100%	0	100	3521682	CP106977.1
Phytophthora agathidicida strain 3770 chromosome 6	*Phytophthora agathidicida*	863	45323	100%	0	100	4894808	CP106976.1
Phytophthora agathidicida strain 3770 chromosome 5	*Phytophthora agathidicida*	863	46740	100%	0	100	5717954	CP106975.1
Phytophthora agathidicida strain 3770 chromosome 4	*Phytophthora agathidicida*	863	56632	100%	0	100	7450011	CP106974.1
Phytophthora agathidicida strain 3770 chromosome 3	*Phytophthora agathidicida*	863	42188	100%	0	100	8108413	CP106973.1
Phytophthora agathidicida strain 3770 chromosome 2	*Phytophthora agathidicida*	863	59631	100%	0	100	8780368	CP106972.1
Phytophthora agathidicida strain 3770 chromosome 1	*Phytophthora agathidicida*	863	70668	100%	0	100	10534528	CP106971.1
Phytophthora agathidicida strain 3770 chromosome 9	*Phytophthora agathidicida*	854	26880	100%	0	99.58	2539455	CP106979.1
Phytophthora agathidicida strain 3770 chromosome 10	*Phytophthora agathidicida*	849	18482	100%	0	99.37	2186599	CP106980.1

Supplementary Table 3. Transposon Hit table. Hits from BLAST search (blastn; nr/nt) of LTR sequence.

Description	Scientific Name	Max Score	Total Score	Query Cover	E value	% identity	Acc. Len	Accession
Phytophthora agathidicida strain 3770 chromosome 5	*Phytophthora agathidicida*	15565	3.05E+05	100%	0	99.99	5717954	CP106975.1
Phytophthora agathidicida strain 3770 chromosome 4	*Phytophthora agathidicida*	15565	4.14E+05	100%	0	99.99	7450011	CP106974.1
Phytophthora agathidicida strain 3770 chromosome 3	*Phytophthora agathidicida*	15565	2.49E+05	100%	0	99.99	8108413	CP106973.1
Phytophthora agathidicida strain 3770 chromosome 1	*Phytophthora agathidicida*	15565	5.00E+05	100%	0	99.99	1053452 8	CP106971.1
Phytophthora agathidicida strain 3770 chromosome 7	*Phytophthora agathidicida*	15556	1.93E+05	100%	0	99.97	3521682	CP106977.1
Phytophthora agathidicida strain 3770 chromosome 6	*Phytophthora agathidicida*	15556	3.26E+05	100%	0	99.97	4894808	CP106976.1
Phytophthora agathidicida strain 3770 chromosome 2	*Phytophthora agathidicida*	15547	4.04E+05	100%	0	99.94	8780368	CP106972.1
Phytophthora agathidicida strain 3770 chromosome 10	*Phytophthora agathidicida*	15431	1.43E+05	100%	0	99.59	2186599	CP106980.1
Phytophthora agathidicida strain 3770 chromosome 8	*Phytophthora agathidicida*	15399	1.31E+05	100%	0	99.47	3225653	CP106978.1
Phytophthora agathidicida strain 3770 chromosome 9	*Phytophthora agathidicida*	15366	1.58E+05	100%	0	99.33	2539455	CP106979.1
Phytophthora sojae hypothetical protein partial mRNA	*Phytophthora sojae*	2091	2197	29%	0	78.88	2873	XM_009526011. 1
Phytophthora plurivora strain TJ71 chromosome 13	*Phytophthora plurivora*	2003	5966	45%	0	77.2	5184793	CP125268.1
Phytophthora plurivora strain TJ71 chromosome 12	*Phytophthora plurivora*	1976	8038	53%	0	77.64	2241191	CP125267.1
Phytophthora plurivora strain	*Phytophthora plurivora*	1973	2576	38%	0	77.78	2097518	CP125264.1

TJ71 chromosome 9								
Phytophthora plurivora strain TJ71 chromosome 16	*Phytophthora plurivora*	1962	3707	39%	0	77.5	1059000	CP125271.1
Phytophthora cinnamomi CRN effector protein (CRN50) gene, partial cds	*Phytophthora cinnamomi*	1953	2815	51%	0	76.79	11508	OR501742.1
Phytophthora ramorum transposon GypsyPr-2 reverse transcriptase gene, partial cds	*Phytophthora ramorum*	1930	2092	34%	0	76.22	3750	DQ645744.1
Phytophthora plurivora strain TJ71 chromosome 2	*Phytophthora plurivora*	1929	3279	38%	0	76.98	3163716	CP125257.1
Phytophthora plurivora strain TJ71 chromosome 11	*Phytophthora plurivora*	1926	2506	37%	0	77.28	2324077	CP125266.1
Phytophthora plurivora strain TJ71 chromosome 1	*Phytophthora plurivora*	1898	9807	48%	0	76.06	6640950	CP125256.1
Phytophthora plurivora strain TJ71 chromosome 10	*Phytophthora plurivora*	1761	5909	50%	0	76.82	2652796	CP125265.1
Phytophthora plurivora strain TJ71 chromosome 4	*Phytophthora plurivora*	1324	2686	36%	0	77.03	1522004	CP125259.1
Phytophthora infestans strain 220 gypsy-like retrotransposon , partial sequence	*Phytophthora infestans*	1266	1531	23%	0	73.74	5749	AF490339.1
Phytophthora plurivora strain TJ71 chromosome 5	*Phytophthora plurivora*	853	2378	30%	0	77.16	2796520	CP125260.1
Phytophthora sojae transposon GypsyPs-2 reverse transcriptase gene, partial cds	*Phytophthora sojae*	828	828	11%	0	78.29	1446	DQ645741.1
Phytophthora infestans gypsy-like retrotransposon	*Phytophthora infestans*	570	937	26%	8.00E -160	69.77	8977	AY830104.1

GypsyPi-3a, complete sequence								
Phytophthora infestans gypsy-like retrotransposon GypsyPi-1d, complete sequence	*Phytophthora infestans*	526	685	21%	9.00E -147	69.94	8252	AY830103.1
Phytophthora plurivora strain TJ71 chromosome 7	*Phytophthora plurivora*	520	674	23%	4.00E -145	69.14	3231242	CP125262.1

Supplementary Table 4. Calculations for estimated weight of diploid genome.

P. agathidicida genome	57 million base pairs
Diploid *P. agathidicida* genome	114 million base pairs
Weight of one base pair	660 Da
	1.0959×10^{-6} fg
Weight of diploid genome	1.0959×10^{-6} fg x 114,000,000
	125 fg

References

Cox, M. P., Guo, Y., Winter, D. J., Sen, D., Cauldron, N. C., Shiller, J., Bradley, E. L., Ganley, A. R., Gerth, M. L., Lacey, R. F., McDougal, R. L., Panda, P., Williams, N. M., Grunwald, N. J., Mesarich, C. H., & Bradshaw, R. E. (2022). Chromosome-level assembly of the *Phytophthora agathidicida* genome reveals adaptation in effector gene families. *Frontiers in Microbiology*, *13*, 1038444. https://doi.org/https://doi.org/10.3389/fmicb.2022.1038444

Klymus, K. E., Merkes, C. M., Allison, M. J., Goldberg, C. S., Helbing, C. C., Hunter, M. E., Jackson, C. A., Lance, R. F., Mangan, A. M., Monroe, E. M., Piaggio, A. J., Stokdyk, J. P., Wilson, C. C., & Richter, C. A. (2020). Reporting the limits of detection and quantification for environmental DNA assays. *Environmental DNA*, *2*(3), 271-282. https://doi.org/https://doi.org/10.1002/edn3.29

McDougal, R., Bellgard, S., Scott, P., & Ganley, B. (2014). Comparison of a real-time PCR assay and a soil bioassay technique for detection of *Phytophthora* taxon *Agathis* from soil. Kauri Dieback response, MPI contract report 53789. New Zealand Forestry Research Institute Ltd. [https://www.kauridieback.co.nz/media/1632/2014-17101-real-time-pcr-as-diagnostic-tool-comparison-of-a-real-time.pdf].

Than, D. J., Hughes, K. J. D., Boonhan, N., Tomlinson, J. A., Woodhall, J. W., & Bellgard, S. E. (2013). A TaqMan real-time PCR assay for the detection of *Phytophthora* 'taxon *Agathis'* in soil, pathogen of Kauri in New Zealand. *Forest Pathology*, *43*(4), 324-330. https://doi.org/https://doi.org/10.1111/efp.12034

Winkworth, R. C. (2020). A LAMP at the end of the tunnel: a rapid, field deployable assay for the kauri dieback pathogen, *Phytophthora agathidicida*. *PLoS One*, *15*(1), e0224007. https://doi.org/https://doi.org/10.1371/journal.pone.0224007
